# Expression of quiescin sulfhydryl oxidase 1 is associated with a highly invasive phenotype and correlates with a poor prognosis in Luminal B breast cancer

**DOI:** 10.1186/bcr3407

**Published:** 2013-03-28

**Authors:** Benjamin A Katchman, I Tolgay Ocal, Heather E Cunliffe, Yu-Hui Chang, Galen Hostetter, Aprill Watanabe, Janine LoBello, Douglas F Lake

**Affiliations:** 1 School of Life Sciences, Arizona State University, PO Box 874501, Tempe, AZ 85287-4501, USA; 2 Department of Laboratory Medicine and Pathology, Mayo Clinic Arizona, 13400 E. Shea Blvd., Scottsdale, AZ 85259, USA; 3 Department of Investigational Pathology, Translational Genomics Research Institute, 445 N Fifth St., Phoenix, AZ 85004, USA; 4 Division of Health Sciences Research, Mayo Clinic Arizona, 13208 E. Shea Blvd., Scottsdale, AZ 85259, USA

## Abstract

**Introduction:**

Quiescin sulfhydryl oxidase 1 (*QSOX1*) oxidizes sulfhydryl groups to form disulfide bonds in proteins. Tumor specific expression of *QSOX1 *has been reported for numerous tumor types. In this study, we investigate *QSOX1 *as a marker of breast tumor progression and evaluate the role of *QSOX1 *as it relates to breast tumor growth and metastasis.

**Methods:**

Correlation of *QSOX1 *expression with breast tumor grade, subtype and estrogen receptor (ER) status was gathered through informatic analysis using the "Gene expression based Outcome for Breast cancer Online" (GOBO) web-based tool. Expression of *QSOX1 *protein in breast tumors tissue microarray (TMA) and in a panel of breast cancer cell lines was used to confirm our informatics analysis. To investigate malignant cell mechanisms for which *QSOX1 *might play a key role, we suppressed *QSOX1 *protein expression using short hairpin (sh) RNA in ER+ Luminal A-like MCF7, ER+ Luminal B-like BT474 and ER- Basal-like BT549 breast cancer cell lines.

**Results:**

GOBO analysis revealed high levels of *QSOX1 *RNA expression in ER+ subtypes of breast cancer. In addition, Kaplan Meyer analyses revealed *QSOX1 *RNA as a highly significant predictive marker for both relapse and poor overall survival in Luminal B tumors. We confirmed this finding by evaluation of *QSOX1 *protein expression in breast tumors and in a panel of breast cancer cell lines. Expression of *QSOX1 *in breast tumors correlates with increasing tumor grade and high Ki-67 expression. Suppression of *QSOX1 *protein slowed cell proliferation as well as dramatic inhibition of MCF7, BT474 and BT549 breast tumor cells from invading through Matrigel™ in a modified Boyden chamber assay. Inhibition of invasion could be rescued by the exogenous addition of recombinant *QSOX1*. Gelatin zymography indicated that *QSOX1 *plays an important role in the function of MMP-9, a key mediator of breast cancer invasive behavior.

**Conclusions:**

Taken together, our results suggest that *QSOX1 *is a novel biomarker for risk of relapse and poor survival in Luminal B breast cancer, and has a pro-proliferative and pro-invasive role in malignant progression partly mediated through a decrease in MMP-9 functional activity.

## Introduction

Breast adenocarcinoma is the most common cancer diagnosed in women throughout the world [1]. In 2012, an estimated 226,870 new cases of invasive breast cancer are expected to occur among US women, and an estimated 39,510 breast cancer deaths [2,3]. Despite significant advances in subtype classification of breast cancers, context-specific drivers of invasion and metastasis are still poorly understood. Our laboratory has focused on defining tumor-specific expression of proteins predicted to play an important role in malignant tumor biology. Recently our lab reported the identification of a short peptide that maps back to the C-terminus of *QSOX1 *in plasma from pancreatic cancer patients [4]. Subsequently, we found that *QSOX1 *is over-expressed in tumor tissue from pancreatic cancer patients, but not adjacent normal tissue [5]. *In vitro *studies with pancreatic cancer cells determined that *QSOX1 *plays a significant role in pancreatic tumor cell growth and metastatic potential. To determine if *QSOX1 *overexpression may be functionally relevant in other tumor types we performed immunohistochemistry (IHC) on breast tissue microarrays and discovered that the expression of *QSOX1 *is specific to malignant breast tumors as well, and has diagnostic and prognostic significance in publicly available microarray datasets. These findings led us to hypothesize that over-expression of *QSOX1 *might be functionally conserved between pancreatic ductal adenocarcinoma and breast adenocarcinoma, prompting further exploration of the potential malignant function of *QSOX1*.

*QSOX1 *belongs to the family of FAD-dependent sulfhydryl oxidases with expression in all sequenced eukaryotic organisms to date, indicating that *QSOX1 *provides a significant and highly conserved function among organisms. The primary enzymatic function of *QSOX1 *is oxidation of sulfhydryl groups, generating disulfide bonds in proteins, ultimately reducing oxygen to hydrogen peroxide [6-8]. Previous work has reported the localization of *QSOX1 *to the Golgi apparatus and endoplasmic reticulum in human embryonic fibroblasts where it works independently as well as with protein disulfide isomerase to help fold nascent proteins in the cell [9-11].

In humans, *QSOX1 *is located on chromosome 1q24 and alternative splicing generates a long (*QSOX1*-L) and short (*QSOX1*-S) transcript [12]. Both, *QSOX1*-S and -L have identical functional domain organization, although *QSOX1*-L contains a predicted transmembrane domain that is not present in *QSOX1*-S due to alternative splicing in exon 12 [12]. While the majority of research to date has focused on the sulfhydryl oxidase activity of *QSOX1 *to efficiently generate disulfide bonds in proteins [8,13,14], the major biological substrates of *QSOX1 *and the functional significance associated with each *QSOX1 *splice variant remain elusive.

Evidence supporting a pro-malignant role for *QSOX1 *expression has also been reported in prostate tumor cells by Song and colleagues [15]. Using knockdown studies they were able to show that the loss of NKX3.1, a transcription factor that is absent in 80% of metastatic prostate cancers, dramatically increased expression of *QSOX1 *in early stages of prostatic neoplasia and throughout the progression of invasive prostate cancer, but was not shown to be present in the normal prostate [15]. NKX3.1 is a known tumor suppressor that is exclusively expressed in luminal epithelial cells of the prostate. This finding is consistent with our observation of *QSOX1 *over-expression in the pancreas as well as in breast adenocarcinoma [5].

In the present study, we evaluated *QSOX1 *protein expression in breast adenocarcinoma cell lines MCF7, BT474 and BT549 and in a breast tumor tissue microarray. Using short hairpin RNA (shRNA) specific for QSOX1-S and -L, we assessed the effects of *QSOX1 *knockdown on cell growth, cell cycle, apoptosis, invasion and matrix metalloproteinase activity. The loss of *QSOX1 *significantly affected tumor cell proliferation and dramatically suppressed tumor cell invasion through Matrigel™. The addition of exogenous recombinant human *QSOX1 *(rh*QSOX1*) rescued the invasive capabilities of MCF7, BT474 and BT549 validating the pro-invasive function of *QSOX1*. We further report the mechanism of *QSOX1*-mediated invasion *in vitro *is due, in part, to elevated MMP-9 activity.

## Material and methods

### Cell culture

Breast adenocarcinoma MCF7, MDA-MB-468, MDA-MB-453, BT474, ZR75, BT549 and MDA-MB-231 cancer cell lines were cultured in DMEM with 10% fetal bovine serum (FBS) (Life Technologies, Grand Island, New York, USA). Immortal human non-tumorigenic breast epithelial cells (MCF10A) were cultured in Clontech KGM-2 karotinocyte media (Gibco). All cell lines were grown at 37°C with 5% CO_2_.

All cell lines tested negative for mycoplasma contamination using the Venor GeM Mycoplasma Detection Kit, (Sigma-Aldrich, St. Louis, Missouri, USA).

### Immunohistochemistry (IHC) and scoring of staining intensity

Breast tumor microarray slides were generated from 153 different breast cancer patients. Each patient's tumor was represented in triplicate on the slides. Immunohistochemistry on breast tumor tissue microarray samples was performed exactly as previously described [16]. After staining the TMA slides with anti-*QSOX1 *rabbit polyclonal antibody, a board certified pathologist (ITO) scored the staining pattern as i) the percentage of cells with IHC staining for *QSOX1 *protein expression in the core tumor tissue sample (0: no staining, 1 (Low): 1 to 33%, 2 (Intermediate): 34 to 66%, 3 (High): 67 to 100%), and ii) the intensity of the antibody stain (0: no staining, 1: weak, 2: moderate, 3: strong staining intensity).

All samples were pre-existing and de-identified and, therefore, exempt from review by the human subjects Institutional Review Board at Arizona State University.

### Statistical assessment of QSOX1 IHC with molecular subtypes of breast cancer

There were 153 patient tissue samples in triplicate stained with anti-*QSOX1 *rabbit polyclonal Ab (Proteintech, Chicago, Illinois, USA). IHC staining was scored by a board certified pathologist (ITO). The amount and intensity of *QSOX1 *staining/expression was scored on a scale of 0 to 3. The first IHC score number represents the percentage of cells staining (0: No staining, 1: 1 to 33%, 2: 34 to 66%, 3: 67 to 100%), and the second represents intensity (0: No staining, 1: weak, 2: moderate, 3: strong staining intensity). We grouped the scores into four categories: 0 (No staining), 11/12/21 (Low staining), 22/13/31 (Intermediate staining) and 32/33/23 (High staining).

To evaluate the relationship between markers (Tumor grade, Her2, CK5/6, and Ki-67) and *QSOX1*, Pearson's chi-square test was performed. Using two-sided *P-*values, statistical significance will be set at *P *≤ 0.05.

### Generation of short hairpin (sh) RNA and lentiviruses production

Two different shRNA for *QSOX1 *were obtained through DNASU in the lentiviral pLKO.1-puromycin selection vector. QSOX1 sh742, 5' - CCGG*GCCAATGTGGTGAGAAAGTTT*CTCGAGAAACTTTCTCACCACATTGGCTTTTTG - 3' (sense), *QSOX1 *sh528, 5'- *CCGGACAATGAAGAAGCCTTT *- 3' (sense), and shScramble with target sequence 5' -*TCCGTGGTGGACAGCCACATG *- 3' was obtained from Dr. Josh LaBaer's laboratory at Arizona State University. The target sequence is underlined and each vector contains the same supporting sequence surrounding the target sequence. Lentiviruses containing sh742, sh528 and shScramble were produced as previously reported by Katchman *et al*. 2011 [5].

### Generation of shQSOX1-transduced tumor cell lines

Stable transduction of sh742, sh528 and shScramble into MCF7, BT474 and BT549 cell lines was performed by first seeding the cells at 6 × 10^5 ^cells/well in a six-well plate and incubating overnight. The next day the cells were transduced by adding 8 ug/mL polybrene (EMD Millipore Corporation, Billerica, Massachusetts, USA) and 200 ul sh742, sh528 and shScramble lentivirus produced from 293T cells to each well. The cells were then incubated for 24 hours. The following day, fresh DMEM with 10% FBS was added, containing 1 ug/mL puromycin (Sigma) to select for the transduced cells. *QSOX1 *knockdown was measured by Western blot.

### SDS-PAGE-Western blotting

Western blotting was performed using cell lysates from MCF10A, MCF7, MDA-MB-468, MDA-MB-453, BT474, ZR 75, BT549 and MDA-MB-231. Cell lysates were generated by harvesting 2.5 × 10^6 ^cells by centrifugation followed by lysis using RIPA buffer (50 mM Tris-HCl, pH 7.4, 150 mM NaCl, 1 mM EDTA, and 1% Triton X-100) with 1× SigmaFAST Protease Inhibitor Cocktail Tablet, EDTA Free. Protein in the cell lysate was measured using the micro BCA protein assay kit (Thermo Fisher Scientific, West Palm Beach, Florida, USA). All samples were then normalized to 2 mg/mL (20 ug total protein per lane). Samples were run on 10% SDS-polyacrylamide gels then transferred onto Immun-Blot™ PVDF Membranes (Bio-Rad, Hercules, California, USA). Rabbit polyclonal anti-*QSOX1 *(ProteinTech), rabbit polyclonal anti-alpha-tubulin (Cell Signaling), rabbit polyclonal anti-MMP-2 and -9 (Sigma), mouse monoclonal caspase 3 (Cell Signaling Technology, Danvers, Massachusetts, USA), and rabbit polyclonal LC3 (Cell Signaling) antibodies were diluted according to the manufacturer's instructions and as determined in preliminary experiments, in 1% BSA in 1× TBS + 0.01% Tween-20 and incubated overnight. Goat anti-rabbit or anti-mouse IgG-alkaline phospatase or HRP secondary antibody was used at a 1:5,000 dilution and incubated with the blot for 1 h followed by washing. BCIP/NBT substrate (Pierce Chemical, Rockford, IL, USA) was added and the blot was developed at room temperature (RT) for approximately 10 minutes for alkaline phosphatase secondary antibody. For samples incubated in goat anti-rabbit or mouse HRP secondary antibody, the blots were developed using Novex ECL Chemiluminescent Substrate Reagent Kit (Novex Life Technologies, Grand Islandm New York, USA). Quantification of band intensity was measured using Image J (Abramoff, M.D., Magalhaes, P.J., Ram, S.J. "Image Processing with ImageJ". Biophotonics International, volume 11, issue 7, pp. 36-42, 2004) and is presented as percent change from the scrambled shRNA control. Full gel images are available in the Additional file 1. All gel images were annotated and processed using Adobe Photoshop CS3 (Adobe Systems Incorporated, San Jose, California, USA).

### MTT (3-(4,5-Dimethylthiazol-2-yl)-2,5-diphenyltetrazolium bromide) assay

Cells were seeded at 3 × 10^3 ^cells/well in a 96-well plate in triplicate and incubated at 37°C, 5% CO_2 _over the course of five days. The MTT assay was performed over a five-day period according to the manufacturer's instructions (Life Technologies Invitrogen-Molecular Probes, Vybrant MTT Cell Proliferation Assay Kit, Grand Island, New York, USA). Results are presented as mean +/- S.D. Student's two tailed *t*-test was performed to determine significance.

### Trypan Blue live/dead cell growth assay

Cells were seeded at 2.5 × 10^4 ^cells/well in a 12-well plate in triplicate and incubated at 37°C, 5% CO_2 _over the course of five days. The cells were removed with Cell Stripper, pelleted and brought back up in 1 mL PBS. A 30 ul aliquot was then used to determine total cell number. The cells were stained at a 1:1 ratio with 0.1% Trypan Blue and are reported as total number of live cells.

### RNA Isolation and cDNA Synthesis

Total RNA isolation was performed according to the manufacturer's instructions for animal cells using spin technology (RNeasy Mini Kit, Qiagen, Gaithersburg, Maryland, USA). After RNA was isolated from each sample was reverse transcribed with qScript cDNA Sythesis Kit (Quanta Biosciences, Gaithersburg, Maryland, USA) according to the manufacturer's instructions.

### Quantitative Real Time PCR (qPCR)

The relative level of GAPDH, *QSOX1*-L, *QSOX1*-S, MMP-2 and MMP-9 were analyzed in each sample by qPCR. Each cDNA sample was normalized to 100 ng/μl in molecular grade water along with 100 nM final concentration of each primer and 1× final concentration of PerfeCta SYBR Green Fast Mix (Quanta Biosciences, Gaithersburg, Maryland, USA), ROX to a final volume of 10 μl. qPCR was performed using PerfeCTa SYBR Green FastMix, ROX from Quanta Biosciences (Quanta Biosciences, Gaithersburg, Maryland, USA) on an ABI7900HT thermocycler, Applied Biosystems Inc. (Life Technologies, Grand Island, New York, USA) Reaction protocol: initial denaturation was as follows - 95°C for 3 minutes; PCR Cycling (40 cycles) 1.) 95°C, 30 sec. 2.) 55°C, 30 sec. 3.) 72°C, 1 minute; Melt Curve (Dissociation stage). The primer sequences for the genes analyzed are: GAPDH Forward 5' - GGCCTCCAAGGAGTAAGACC; GAPDH Reverse 5' - AGGGGTCTACATGGCAACTG; *QSOX1*-S Forward 5' - TGGTCTAGCCACAACAGGGTCAAT; *QSOX1*-S Reverse 5' - TGTGGCAGGCAGAACAAAGTTCAC; *QSOX1*-L Forward 5' - TTGCTCCTT GTCTGGCCTAGAAGT; *QSOX1*-L Reverse 5'-TGTGTCAAAGGAGCTCTCTCTGTCCT; MMP-2 Forward 5' - TTGACGGTAAGGACGGACTC; MMP-2 Reverse 5' - ACTTGCAGTACTCCCCATCG; MMP-9 Forward 5' - TTGACAGCGACAAGAAGTGG; MMP-9 Reverse 5' - CCCTCAGTGAAGCGGTACAT. Each reaction was performed in triplicate with the data representing the averages of one experiment.

In the shRNA experiment, expression of MMPs was normalized to the non-targeted GAPDH to determine ΔCq. ΔCq replicates were then exponentially transformed to the ΔCq expression after which they were averaged ± standard deviation. The average was then normalized to the expression of the shScramble control to obtain the ΔΔCq expression. Significance was determined using the Student's two tailed *t*-test.

### Boyden chamber and invasion recovery assay

Invasion assays were performed using BD BioCoat™ BD Matrigel™ and non-Matrigel™ control invasion chambers (BD Biosciences, San Jose, California, USA) with 8.0 μm pore size polyethylene terephthalate (PET) membrane inserts in 24-well format. The assay was performed according to the manufacturer's instructions (BD Bioscience, San Jose, California, USA). A total of 4 × 10^4 ^cells/well were seeded into the inner Matrigel™ chamber in serum free DMEM. The outer chamber contained 10% FBS in DMEM. MCF7, BT474 and BT549 cells were incubated for 72, 48 and 48 hours, respectively at 37°C, 5% CO_2_. For invasion rescue assays MCF7, BT474 and BT549, cells were incubated with 50 nM r*QSOX1 *as well as catalytically inactive mutant r*QSOX1 *(r*QSOX1*-AA). Cells that invaded through the Matrigel™ and migrated through the pores onto the bottom of the insert were fixed in 100% methanol and then stained in hematoxylin (Invitrogen-Life Technologies, Grand Island, New York, USA). The total number of invading cells was determined by counting the cells on the underside of the insert from triplicate wells (six fields per insert) at 20× magnification. The extent of invasion was expressed as the mean +/- S.D. Significance was determined using the Student's two-tailed *t*-test. Results presented are from one of three independent experiments.

### Gelatin zymography

The identification of MMP was performed using gelatin zymography. Zymography experiments were performed essentially as previously described by Katchman *et al*. [5]. Minor changes in the protocol are the inclusion of untreated MCF7 and BT549 cells as well as short hairpin-transduced cells were seeded at 5 × 10^5 ^cells/well (12-well plates) in DMEM with 10% FBS. The next day, cells were then washed with 1 × PBS and the media was changed to serum-free DMEM and incubated for 48 hours instead of 24 hours before being collected and protein concentrations determined using a BCA assay. Quantification of band intensity was measured using Image J and is presented as the percent change from the scrambled shRNA control.

## Results

### Expression of QSOX1 correlates with poor prognosis in patients with *Luminal B *breast cancer

**Figure 1:**
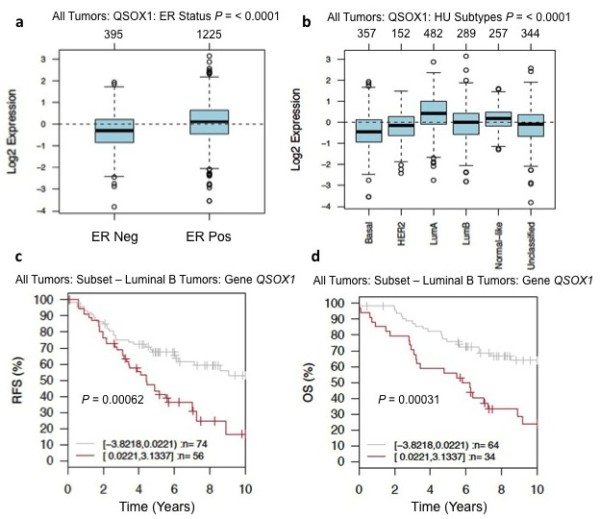
**GOBO analyses of QSOX1 transcript expression among subtypes of breast cancer from over 1,800 cases**. **a) **Box plot analysis of QSOX1 mRNA expression in all tumors ER+ (*n *= 1,225) and ER- tumors (*n *= 395) (*P *< 0.00001); **b) **Box plot analyses of QSOX1 expression among HU subtypes, Basal (*n *= 357), HER2 (*n *= 152), Luminal A (*n *= 482), Luminal B (*n *= 289), Normal-like (*n *= 257) and unclassified (*n *= 344), (*P *< 0.00001). **c) **Kaplan Meier analysis over 10 years of relapse free survival (RFS) in patients with Luminal B breast cancer expressing high (red line) and low (gray line) QSOX1 mRNA; High (*n *= 56), low (*n *= 74), (*P *= 0.00062) and **d) **Overall survival (OS); High (*n *= 34), low (*n *= 64), (*P *= 0.00031). Data obtained using GOBO, Gene expression based Outcome for Breast cancer Online.

Bioinformatic analysis of *QSOX1 *transcript expression was assessed using data from the Gene expression based Outcome for Breast cancer Online algorithm (GOBO) [17]. GOBO is a web based analysis tool that utilizes Affymetrics gene expression data curated from 1,881 breast cancer patients with associated stage, grade, nodal status and intrinsic molecular classification based on the paradigm first reported by the Perou Laboratory [18]. Expression of *QSOX1 *was significantly higher in ER+ tumors compared to ER- (*P*-value < 0.00001), with the highest expression observed in Luminal A, Luminal B and Normal-like subtypes (Figure 1a, b). The lowest *QSOX1 *transcript expression was observed in HER2-enriched and basal tumors. Using the GOBO tool, we performed a series of Kaplan Meier analyses to determine whether *QSOX1 *expression is associated with relapse free survival (RFS) and overall survival (OS) (Figure 1c, d and Additional file 2). While elevated *QSOX1 *expression is not associated with survival when considering all breast tumor subtypes together (see Additional file 2), it is highly associated with poor RFS (*P *= 0.00062) and OS (*P *= 0.00031) in Luminal B tumors (Figure 1c, d). The expression of *QSOX1 *correlates with increasing tumor grade as well as poor overall survival in patients diagnosed with grade 2 (*P *= 0.04242) and grade 3 (*P *= 0.07095) breast tumors (see Additional file 2i-k). Elevated *QSOX1 *was also associated with reduced OS in luminal A tumors (see Additional file 2g, h) and is a predictor of poor OS for patients who did not receive systemic treatment (see Additional file 2d).

### Evaluation of QSOX1 expression by immunohistochemistry

**Figure 2:**
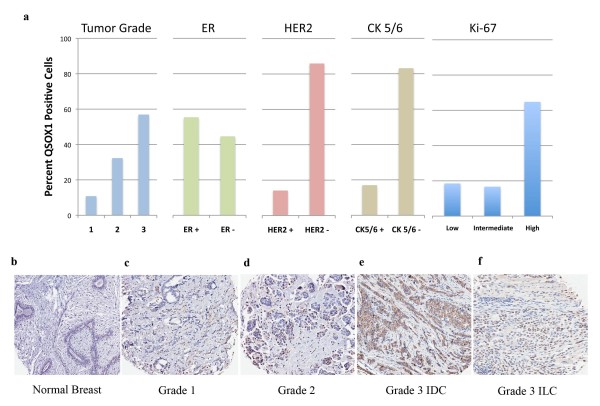
**Protein expression of QSOX1 is specific for breast tumor cells in tissue**. **a.) **Graphical representation of "High QSOX1 staining (*n *= 65)" column from Table 1. Each graph correlates with percentages of QSOX1 positive cells listed in Table 1 for the "High QSOX1 staining (*n *= 65)" column **b.) **normal breast tissue showing no QSOX1 staining; **c.) **grade 1 invasive ductal carcinoma (IDC) ER+ PR+ breast tumor tissue showing low QSOX1 staining; **d.) **grade 2 IDC ER+ PR+ breast tumor tissue showing moderate QSOX1 staining; **e.) **grade 3 IDC, ER+, PR+ showing high QSOX1 staining; **f.) **grade 3 invasive lobular carcinoma (ILC), ER+, PR- showing high QSOX1 staining. Polyclonal antibody recognizes residues 1-329 of both QSOX1-S and -L.

Results from the GOBO transcript expression analysis fueled investigation of *QSOX1 *at the protein level in breast tumors. A breast tumor tissue microarray composed of breast tumors from over 150 different patients was stained with a rabbit anti-*QSOX1 *polyclonal antibody and scored by a board certified pathologist (ITO). Figure 2b shows no expression of *QSOX1 *in normal breast tissue. Figure 2c-f represent a pattern of increasing *QSOX1 *expression observed in the TMA in grade 1, grade 2 and grade 3 invasive ductal carcinomas and a grade 3 invasive lobular carcinoma. Statistical evaluation of *QSOX1 *expression by immunohistochemistry (IHC) demonstrated an association with ER+ tumors, and a strong association with high Ki-67 expression in patients with a high *QSOX1 *IHC score (Figure 2a, Table 1). There was no relationship observed for *QSOX1 *expression in HER2+ tumors or cytokeratin markers (CK 5/6) positive tumors. These data are consistent with the correlation observed in the GOBO data. Interestingly, higher grade tumors were associated with a higher *QSOX1 *IHC score (Figure 2a, Table 1). Conversely, lower *QSOX1 *protein expression is significantly associated with lower grade tumors. This is consistent with an association between *QSOX1 *expression and more aggressive ER+ tumors.

**Table 1:** Statistical assessment of QSOX1 protein expression with molecular subtypes of breast cancer

		IHC Score			*P*-value
		
	No QSOX1 staining (*n *= 17) %	Low QSOX1 staining (*n *= 47) %	Intermediate QSOX1 staining (*n *= 24) %	High QSOX1 staining (*n *= 65) %	
**Grade**					*0.0003
**1**	53.3	42.2	25	10.8	
**2**	33.3	33.3	41.7	32.3	
**3**	13.3	24.4	33.3	56.9	
**ER**					*0.0013
**ER +**	80	89.1	73.9	55.4	
**ER -**	20	10.9	26.1	44.6	
**HER2**					0.0811
**HER2 +**	11.8	6.4	29.2	14.1	
**HER2 -**	88.2	93.6	70.8	85.9	
**CK5/6**					0.0733
**CK5/6 -**	100	95.7	87.5	83.1	
**CK5/6 +**	0	4.3	12.5	17	
**KI-67**					*0.0011
**Low**	33.3	33.3	41.1	18.5	
**Intermediate**	44.4	45.5	17.7	16.7	
**High**	22.3	21.2	41.2	64.8	
**ER & HER2**					*0.0016
**ER- HER2 -**	13.3	8.7	8.7	35.9	
**Others**	86.7	91.3	91.3	64.1	
**ER, HER2 and CK5/6**					0.0923
**ER- HER2-, CK5/6: 1/2/3**	0	4.3	4.2	15.4	
**Others**	100	95.7	95.8	84.6	

QSOX1 expression was grouped into four categories based on the percentage of cells stained and the intensity of QSOX1 expression: No expression, Low QSOX1 expression, Moderate QSOX1 expression and Strong QSOX1 expression (see material and methods for detailed explanation). Each number represents the percentage of QSOX1 positive or negative cells within each molecular subtype of breast cancer (*n *= total number of tissue samples within each category). Pearson's chi-square test was performed to determine a relationship between the molecular subtypes and QSOX1 expression. Statistical significance using a two-sided *P-*value was set at *P *≤0.05*.

### Evaluation of QSOX1 expression by Western blot

**Figure 3:**
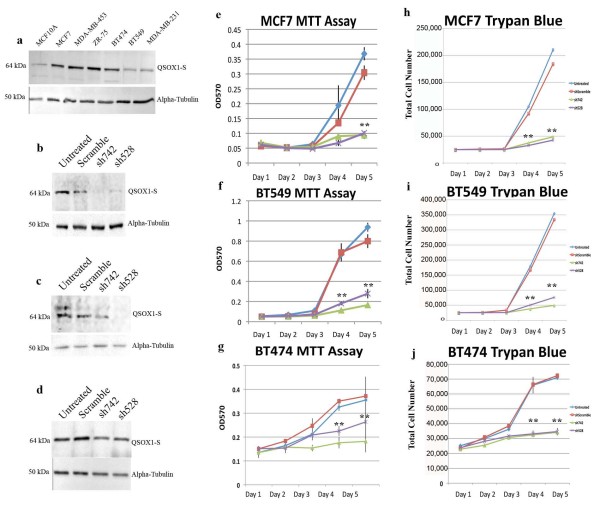
**Reduced expression of QSOX1 leads to a significant decrease in tumor cell growth**. **a) **Western blot showing weak expression of QSOX1 in transformed, but non-tumor-forming MCF10A and human breast ductal carcinoma cell lines Luminal A like (MCF7 and MDA-MB-453), Luminal B like (ZR75 and BT474) and Basal like (BT549 and MDA-MB-231). α-Tubulin loading control is shown below each lane. MCF7, BT549 and BT474 breast tumor cell lines were transduced with lentiviral shRNA QSOX1 (sh742 and sh528). Western blots are shown using the same anti-QSOX1 polyclonal Ab as in Figure 2 on cell lysates from **b.) **MCF7 (percent decrease in sh742: 85% and sh528: 82%); **c.) **BT549 (percent decrease in sh742: 45% and sh528: 77%) and **d.) **BT474 (percent decrease in sh742: 40% and sh528 36%) cells. Western blots have been cropped and full images can be viewed in Additional file 1. **(e-g) **MTT and **(h-j) **Trypan Blue growth assays on MCF7, BT549 and BT474 cells transduced with shScramble, sh742 and sh528 assayed on Days 1 through 5. Percent decrease sh742 and sh528 day 5: **e.) **66% (both); **f.) **78% and 69%; **g.) **52% and 34%; **h.) **72% and 73%; **i.) **98% and 96%; **j.) **50% (both). Experiments were performed three times in triplicate; error bars represent SD from triplicate wells. Significance **, *P *< 0.01.

*QSOX1 *expression in human breast adenocarcinoma was assessed in six different breast tumor cell lines, and a transformed non-tumorigenic breast cell line, MCF10A [19,20]. Similar to our previous studies in pancreas cancer [5], the short form of *QSOX1 *is expressed as the predominant splice variant in each cell line examined (Figure 3a). Consistent with the GOBO and IHC expression data, we found that the expression of *QSOX1*-S protein was more highly expressed in luminal-like cell lines MCF7 (ER+), MDA-MB-453 (ER-), ZR 75 (ER+) and BT474 (ER+) compared to basal-like BT549 and MDA-MB-231 cell lines. Interestingly, *QSOX1 *was most weakly expressed in transformed normal MCF10A cells which do not form tumors in immunodeficient animals.

### Expression of QSOX 1 in tumor cells promotes cellular proliferation

To begin to assess the mechanistic role that *QSOX1 *plays in tumor cells we stably knocked-down *QSOX1 *expression in MCF7, BT549 and BT474 cells using two lentiviral shRNA constructs, sh742 and sh528 (Figure 3b-d). *QSOX1 *protein expression was assessed following stable knock-down relative to isogenic parental cell lines by Western blotting. Densitometry of the *QSOX1 *protein relative to alpha-tubulin expression indicates that sh742 and sh528 resulted in a knock-down of *QSOX1*-S expression in MCF7 cells by 85% and 82%, respectively. In BT549 cells the knock-down was 65% and 77%, and for BT474 cells by 40% and 36%, respectively (Figure 3b-d).

The growth rates of sh*QSOX1*-transduced MCF7, BT549 and BT474 cells were then evaluated compared to isogenic controls (Figure 3e-j). An equal number of untransduced (parental), shScramble, sh742 and sh528 cells were seeded in 96-well plates and assayed for proliferation over five days using the MTT assay. Sh*QSOX1*-transduced MCF7, BT549 and BT474 cells displayed a decrease in cell growth compared to shScrambled and parental controls (Figure 3e-g). In MCF7 cells, sh742 and sh528 showed a 66% decrease in cell growth, while sh742 and sh528 suppressed growth of BT549 by 78% and 69%, respectively, and sh742 and sh528 suppressed growth of BT474 by 52% and 29%, respectively by Day 5. We confirmed our MTT results by performing Trypan Blue staining over five days (Figure 3h-j) using the same incubation conditions as in the MTT assay. These results suggest that *QSOX1 *helps drive tumor cell growth.

### Cell cycle, apoptosis and autophagy analysis

In non-tumor fibroblasts, expression of *QSOX1 *was correlated with the quiescent stage, G_o_, of the cell cycle and overexpression of *QSOX1 *was shown to protect MCF7 cells for ROS mediated apoptosis [21], this led us to hypothesize that a sh*QSOX1*-mediated decrease in cell proliferation could be the result of abnormal regulation of the cell cycle, an increase in apoptosis or the result of autophagosome formation. To address this, propidium iodide (PI) was used in flow cytometry to evaluate the effects of sh*QSOX1 *on cell cycle. In MCF7 cells, both sh*QSOX1 *RNAs showed a slight decrease in G_1 _and an increase (11 to 12%) in S phase, while in BT474 cells both sh*QSOX1 *RNAs showed a slight 12% increase in G_1 _and a 26% decrease in S phase but neither sh*QSOX1 *RNA sequence had any effect in BT549 cells compared to untreated and shScramble controls (see Additional file 3a-c).

Next we determined if the decrease in cellular proliferation was due to an increase in apoptosis or autophagy (see Additional file 3d, e). To assess apoptosis, we analyzed MCF7 and BT474 transduced cells for Annexin V/PI [22]. We subsequently probed MCF7 and BT549 transduced cells for LC3, a protein that is necessary for autophagosome formation [23]. If the expression of *QSOX1 *prevented cellular apoptosis or autophagy we would expect to see an increase in expression of Annexin V and LC3 in sh*QSOX1 *transduced cells, but we did not observe any statistically significant increases in Annexin V positive cells (see Additional file 3d-f). This correlates with our previous results in pancreas cancer that the suppression of *QSOX1 *does not lead to cell death or autophagy.

### Suppression of QSOX1 expression inhibits tumor cell invasion

**Figure 4:**
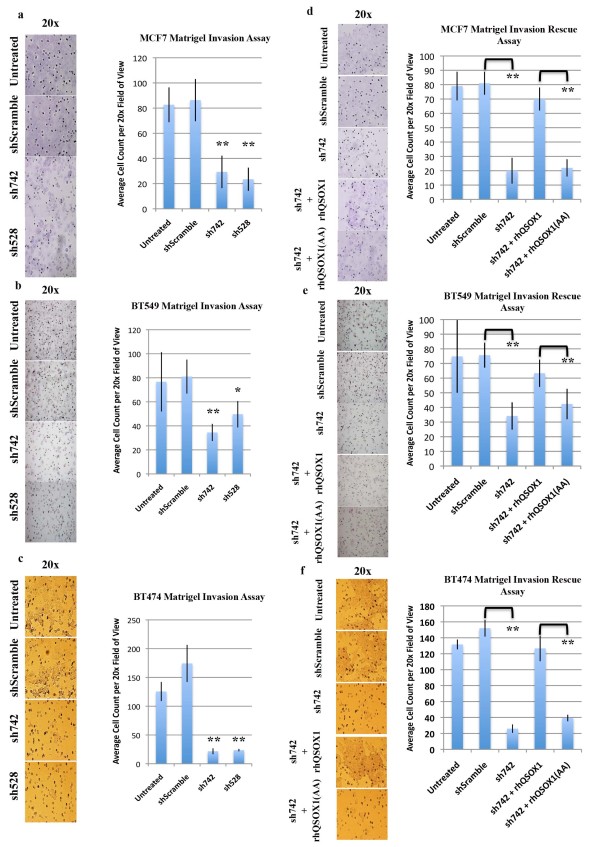
**QSOX1 promotes tumor cell invasion**. **a.) **MCF7, **b.) **BT549 and **c.) **BT474 cells transduced with shSramble, sh742 and sh528 shRNAs were seeded at equal densities in the top chamber of Matrigel™ invasion wells and allowed to incubate for 48 (BT549 and BT474) and 72 (MCF7) hours, after which cells that had digested Matrige^lTM ^and migrated through the 8 um pores were counted on the underside of the insert. Representative 20× images are presented. MCF7 cells transduced with sh742 and sh528 show a 65% and 71% decrease in invasion. BT549 cells transduced with sh742 and sh528 showed a 60% and 40% decrease in invasion. BT474 cells transduced with sh742 and sh528 show an 82% decrease in invasion. Each knockdown was compared to shScramble controls. The invasive phenotype of shQSOX-transduced MCF7 **(d.)**, BT549 **(e.) **and BT474 **(f.) **cells was rescued by exogenous incubation with catalytically active rhQSOX1. rhQSOX1 (AA) mutant is a mutant without enzymatic activity, generously provided by Dr. Debbie Fass. Graphs represent average ± SD (MCF7, BT549 and BT474 *n *= 3), significance *, *P *< 0.05, ** *P *< 0.005.

The process of tumor cell invasion involves the degradation of basement membrane (BM) components such as laminin, collagen and fibronectin before a tumor cell is able to invade other tissues [24]. We performed a modified Boyden chamber assay using Matrigel™-coated inserts in which tumor cells must degrade the Matrigel™ and migrate through a membrane with 8 um pores to gain access to nutrient rich media. Sh742 and sh528-transduced MCF-7, BT549 and BT474 tumor cells were added to Matrigel-coated, 8 um pore inserts in serum-free medium. After 72 (MCF7) and 48 (BT549 and BT474) hours of incubation, tumor cells that were able to degrade Matrigel™ and migrate through 8 um pores onto the underside of the insert were counted (Figure 4a-c). Our results demonstrate that knockdown of *QSOX1 *expression in MCF7 leads to a 65% and 71% reduction in invasion of sh742 and sh528 transduced tumor cells, respectively. For BT549 sh742 and sh528 - transduced tumor cells, 60% and 40% decreases in invasion through Matrigel™ were observed. Suppression of *QSOX1 *expression in BT474 cells leads to an 85% reduction in invasion of both sh742 and sh528 transduced tumor cells. These data suggest that *QSOX1 *plays a role in regulating invasive behavior *in vitro *irrespective of breast tumor subtype and hormone receptor status.

To prove that suppression of *QSOX1 *protein expression was responsible for loss of tumor cell invasion, we performed a rescue experiment in which recombinant human *QSOX1 *(rh*QSOX1*, generously provided by Dr. Colin Thorpe) was added to sh*QSOX1*-MCF7, sh*QSOX1*-BT549 and shQSOX1-BT474 cells during the invasion assay. As a control for the enzymatically active *QSOX1*, a mutant rh*QSOX1 *in which the CxxC motif in the thiredoxin-1 domain was mutated to AxxA (rh*QSOX1*(AA), generously provided by Dr. Debbie Fass) was added to the invasion assay. Addition of enzymatically active rhQSOX1 rescued the invasive phenotype of the sh*QSOX1*-transduced tumor cells (Figure 4d-f), while the addition of the rh*QSOX1*(AA) did not rescue invasion of the sh*QSOX1*-transduced tumor cells.

### Decrease in QSOX1 leads to a decrease in matrix metalloproteinase activity

Since knockdown of *QSOX1 *resulted in decreased breast tumor cell invasion, it was important to determine a mechanism for how *QSOX1 *might facilitate invasion. Matrix metalloproteinases (MMP) have been shown to play key roles in breast tumor invasion and metastasis [24]. Both MMP-2 and -9 mRNA and protein levels have been shown to contribute to breast tumor invasion, metastasis and angiogenesis [25]. Since previous work demonstrated that *QSOX1*-S is secreted into the extracellular matrix where MMPs are activated, we hypothesized that *QSOX1 *might help activate MMP-2 and -9 proteins. MCF7 and BT549 cells transduced with shScramble, sh742 and sh528 were plated at equal densities and allowed to incubate in serum free media for 48 hours, after which the supernatants were collected and analyzed by gelatin zymography to determine if the loss of *QSOX1 *leads to a decrease in the functional activity of MMP-2 and -9.

Initial analysis of the results indicates that MCF7 and BT549 possess similar MMP profiles even though it is known that BT549 cells are more invasive. Luminal B-like breast tumor cell lines BT474 and ZR75 do not secrete detectable levels of MMPs [25,26]. However, both MCF7 and BT549 supernatants contain MMP-9 homodimer (130 kDa), a large amount of proteolytically active pro-MMP-9 (92 kDa) with lesser concentrations of proteolytically active pro-MMP-2 (72 kDa).

**Figure 5:**
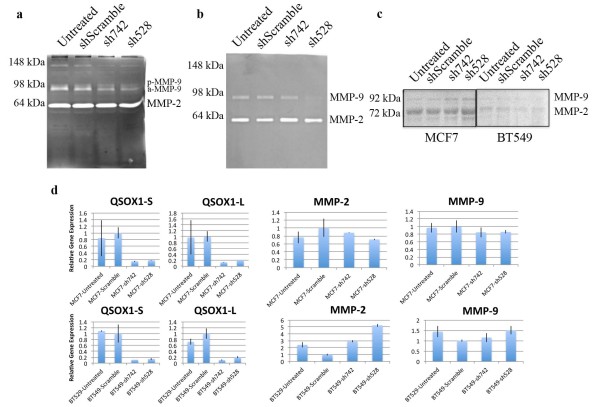
**Reduced expression of QSOX1 in MCF7 and BT549 cells leads to a decrease in functional MMP-9 activity**. Gelatin zymography of **a) **MCF7 and **b) **BT549 conditioned media shows a decrease in MMP-9 homodimers (130 kDa) and MMP-9 (92 kDa). The percent decrease in MMP-9 expression in MCF7 was: sh742: 70% (*P *= 0.0171); sh528: 77% (*P *= 0.0182), and in BT549 was: sh742: 34% (*P *= 0.0531); sh528: 88% (*P *= 0.0564) compared to shScramble control. **c) **Western blots of total cell lysate from shRNA treated MCF7 and BT549 probing for MMP-2 and -9 show insignificant changes compared to shScramble control. Full images can be seen in Additional file 1. **d) **QPCR of QSOX1 transcripts and MMP-2 and -9 transcripts. The graph represents relative gene expression calculated as ΔΔC_q _using *GAPDH *as the endogenous reference gene. MMP-2 - MCF7 sh742 (*P *= 0.5294), sh528 (*P *= 0.2112); BT549 sh742 (*P *= 0.0054), sh528 (*P *= 0.0019). MMP-9 - MCF7 sh742 (*P *= 0.3981), sh528 (*P *= 0.3385); BT549 sh742 (*P *= 0.4192), sh528 (P = 0.0701). Average ± SD; significance was determined using a Student's two-tailed *t*-test.

We found that supernatants from MCF7 cells transduced with sh742 and sh528 showed a 70% and 77% decrease, respectively, in pro-MMP9 activity compared to shScramble (Figure 5a). MCF7 supernatants from cells transduced with sh742 and sh528 also showed a 50% and 60% decrease in active MMP-9 (a-MMP-9) as well (Figure 5a). Supernatants from BT549 cells transduced with sh742 and sh528 showed a 34% and 88% decrease, respectively, in MMP-9 (Figure 5b). Decreases in the proteolytic activity of MMP-9, using gelatin as a substrate, provide a mechanism for the sh*QSOX1*-mediated suppression of invasion through Matrigel™.

To extend our hypothesis that *QSOX1 *is activating or modifying MMPs post-translationally, we performed a Western blot on total cell lysate from MCF7 and BT549 transduced cells as well as performed quantitative real time PCR (qPCR) to determine if the loss of *QSOX1 *affected MMP protein and RNA levels (Figure 5c, d). Our results indicate that the intracellular amount of MMP-2 and -9 protein is similar between the untreated, shScramble, sh742 and sh528 samples in MCF7 and BT549 cells (Figure 5c). Figure 5d demonstrates that the loss of *QSOX1 *also has no significant effect on the transcriptional activity of MMP-2 and -9. These results add confidence to our hypothesis that *QSOX1 *is involved in the post-translational activation of MMPs.

## Discussion

*QSOX1 *protein was reported by our laboratory to be over-expressed in tumors from patients diagnosed with pancreatic ductal adenocarcinoma (PDA) [4]. In a subsequent study we reported that expression of *QSOX1 *promotes pancreatic tumor cell growth and invasion [5]. To determine if *QSOX1 *was also over-expressed in breast cancer, a GOBO analysis was performed using data from over 1,800 breast cancer cases. A prominent finding in this analysis is that the highest levels of *QSOX1 *expression in Luminal B breast cancer correlate with very poor RFS and OS (Figure 1c, d; Additional file 2e, f). The median survival in patients with Luminal B breast cancer who over-express *QSOX1 *is approximately four years. The prognostic power of *QSOX1 *expression for RFS and OS increases when Luminal B breast cancer cases are divided into quintiles using the GOBO analysis tool for which patients with the highest fifth expression of *QSOX1 *have RFS of less than two years and OS of less than three years (see Additional file 2e, f). In support of our GOBO analysis, showing that expression of *QSOX1 *is an indicator of poor OS and RFS in Luminal B breast cancer patients, we performed IHC on breast TMA samples. We were able to confirm that expression of *QSOX1 *significantly correlates with ER+ breast tumor (*P *= 0.0013) cells as well as correlating with high Ki-67 expression (*P *= 0.0011), further supporting a role for *QSOX1 *in cellular proliferation (Figure 2a). Additionally, over-expression of QSOX1 mRNA in the GOBO analysis and high levels of protein in IHC correlate with increasing tumor grade in our breast tumor TMA analyses (Figure 2; Table 1; Additional file 2i-k). Expression of *QSOX1 *did not correlate with survival in HER2 enriched tumors, ER- tumors or in tumors subtyped as basal-like. Importantly, in patients who did not receive systemic therapy (presumably due to diagnosis of very early stage disease), *QSOX1 *appears to be a predictor of poor OS (see Additional file 2d). However, this association was not strong until more than five years post diagnosis. These data collectively suggest that *QSOX1 *is associated with highly proliferative ER+ tumors and warrants further preclinical and prospective validation as a diagnostic and prognostic biomarker in ER+ tumors.

Tumor cells in which *QSOX1 *expression was suppressed using shRNAs grew at less than half the rate of shScramble and untreated controls in MCF7, BT549 and BT474 cells (Figure 3e-j). The results of the MTT and Trypan Blue assays confirm our breast TMA findings showing that high expression of *QSOX1 *correlates with high Ki-67 expression. Our attempt to explain the decrease in cell growth by abnormal cell cycle regulation, apoptosis and autophagy suggests that *QSOX1 *is not involved in apoptosis, or autophagy (see Additional file 3d-f), but may marginally affect cell cycle, as we observed a stall in G_1 _and an increase in S phase in MCF7 cells (luminal-like) and an insignificant increase in G_1 _and a decrease in S phase in BT474 (luminal-like) cells compared to shScramble controls (Additional file 3a-c). However, there were no observable changes in BT549 cells (basal-like). These results, combined with our findings in PDA suggest that *QSOX1 *is unlikely to play a significant role in cell cycle. Our analysis of apoptosis and autophagy as a second possible mechanism contributing to the observed decrease in cell growth did not reveal significant increases in Annexin V/PI or LC3 expression (autophagy) in our shRNA treated cells (see Additional file 3d-f). We also did not observe any increases in Trypan Blue positive cells during our cell growth assays compared to our shScramble control (data not shown). At this time, the exact function of *QSOX1 *with respect to tumor cell proliferation remains elusive.

The ability of a tumor cell to invade is one of several hallmarks of cancer [27]. Based on our previous results showing that *QSOX1 *over-expression in pancreas tumor cells contributes to invasion, we hypothesized that the over-expression of *QSOX1 *in breast adenocarcinoma would elicit a similar phenotype. MCF7, BT549 and BT474 cells transduced with *QSOX1 *shRNAs exhibited significant decreases in their ability to degrade basement membrane components and invade through Matrigel™ (Figure 4a-c). MCF7 cells are a poorly invasive, luminal-A like breast cancer cell line, while BT549 (basal-like) and BT474 (Luminal-B like) cells are highly invasive [28,29]. Although the invasive capabilities are dramatically different between these cell lines, *QSOX1 *knock-down suppressed growth and invasion in all cell lines irrespective of the level of *QSOX1 *expression (Figure 2a) and molecular tumor subtype. Addition of exogenous recombinant *QSOX1 *protein to sh*QSOX1 *transduced tumor cells rescued their invasive properties (Figure 4d-f), confirming previous data suggesting that *QSOX1 *is secreted into the extracellular matrix.

These findings indicate the advantage that *QSOX1 *provides to breast and pancreas tumors may be highly conserved and universal among other tumor types. However, one cannot draw this definitive conclusion from the phenotypic behavior of cells cultured in 2D [30,31]. What we can conclude from our human TMA analysis of *QSOX1 *protein expression is that *QSOX1 *is a very specific marker of tumor cells and that the expression of *QSOX1 *correlates with increased proliferation (high Ki-67) and an increase in tumor grade consistent with the characteristics of highly invasive tumors. *QSOX1 *is likely to become functionally relevant when considered not only in specific molecular subcontext (such as ER+ tumor cells), but in specific environmental contexts within the 3D breast tumor microenvironment with the full complement and complex interplay of autocrine and paracrine signaling components known to be important in tumor progression [28,30,32,33].

MMPs are a family of proteases that are involved in the degradation of basement membrane components contributing to tumor cell invasion and proliferation [34]. In breast tumors, gelatinases, MMP-2 and MMP-9 have been shown to play a significant role in growth and metastasis, as their expression is correlated with aggressive forms of breast cancer [25,34,35]. Gelatinases are secreted into the extracellular matrix in their inactive, pro- form where they can be activated through either a cysteine switch or shift in the prodomain mediated by integrins and laminin in basement membranes and structural proteins, such as vimentin [25]. Thiol binding proteins, such as glutathione, have also been shown to help fold and activate MMPs [35]. Our data suggest that MMPs could be one substrate of *QSOX1*. To address this we performed gelatin zymography to assess functional activity MMPs. Our data reveal that knockdown of *QSOX1 *protein expression in both MCF7 and BT549 cells leads to a decrease in MMP-9 functional activity compared to shScramble control (Figure 5a, b). While the functional activity of MMP-2 and -9 was suppressed, mRNA encoding MMP-2 and -9 remained relatively constant in MCF-7 cells and increased in BT549 cells (Figure 5c, d). BT474 cells unfortunately do not express or secrete levels of MMP-2 and -9 detectable by gelatin zymography [25,36]. Interestingly, when we knock down *QSOX1 *in BT474 cells we observe the same phenotypic effects indicating that there are multiple substrates of *QSOX1 *contributing to our observed decrease in cellular proliferation and invasion. Taken together, the data suggest that *QSOX1 *may post-translationally activate MMPs, although this requires further study to be a definitive conclusion. Future proteomic analysis may reveal a wide spectrum of substrates linked to cellular proliferation, basement membrane production and cellular motility connecting the phenotypes observed in this report to *QSOX1 *expression.

*QSOX1 *is expressed during embryonic development in mouse and rat during key migratory stages [37]. This developmental data combined with our results indicating that *QSOX1 *expression facilitates degradation of basement membranes suggests that tumor cells over-express *QSOX1 *to allow them to break down basement membranes and invade into adjacent tissues or into circulation. It will be interesting to assess *QSOX1 *expression in circulating tumor cells. *QSOX1 *expression in Luminal B subtype may help further stratify which tumors are likely to be more aggressive, leading to poor overall survival. Notably from these data we can project that targeting *QSOX1 *irrespective of tumor subtype could help to slow tumor cell proliferation as well as tumor cell invasion. This finding provides another tool for physicians and their patients to decide whether to more aggressively treat patients with Luminal B breast cancer whose tumors express high levels of *QSOX1*.

While this paper was under review and revision, Pernodet *et al*. reported that *QSOX1 *over-expression in breast cancer is associated with a good prognosis. This report creates a discrepancy of the function of QSOX1 in breast cancer. We previously published that *QSOX1 *promotes pancreatic cancer growth and invasion in a manner very similar to the results shown here. We became interested in the role of *QSOX1 *in breast cancer after we employed the publicly available GOBO analysis tool to evaluate *QSOX1 *gene expression among 1,881 cases of molecularly subtyped breast cancer. The GOBO analysis clearly indicates that *QSOX1 *expression predicts a poor prognosis in patients with luminal type and normal-like breast cancer. This initial GOBO analysis fueled our investigation of *QSOX1 *in breast cancer. Unlike the 217 patient dataset used by Pernodet *et al*., GOBO analysis is completely independent, and agrees with our findings that *QSOX1 *is a bad actor in breast cancer. Furthermore, we have shown immunohistochemically that higher grade tumors express more *QSOX1 *protein than lower grade tumors. It will be interesting to determine the true role of *QSOX1 *in breast and other cancers.

## Conclusions

In this study we show for the first time that *QSOX1 *over-expression is associated with features of poor prognosis in patients whose tumors highly express *QSOX1 *and that *QSOX1 *promotes breast tumor growth and invasion *in vitro*, perhaps mediated mechanistically by post-translational activation of MMP-9 functional activity. While further research is still needed to understand the role of *QSOX1 in vivo*, the results presented here strongly suggest that targeted inhibition of *QSOX1 *may stall cancer progression.

## Abbreviations

BM: Basement membrane; BSA: Bovine serum albumin; DMEM: Dulbecco's modified Eagle's medium; ER: Estrogen receptor; FBS: Fetal bovine serum; GOBO: Gene Expression Based Outcome for Breast Cancer Online; IDC: Invasive ductal carcinoma; IHC: Immunohistochemistry; ILC: Invasive lobular carcinoma; MMP: Matrix metalloproteinases; MTT: 3-(4,5-Dimethylthiazol-2-yl)-2,5-diphenyltetrazolium bromide, a yellow tetrazole; OS: Overall survival; PDA: Pancreatic ductal adenocarcinoma; PET: polyethylene terephthalate; PI: Propidium iodide; qPCR: Quantitative real time PCR; QSOX1: Quiescin sulfhydryl oxidase 1; RFS: Relapse free survival; rhQSOX1: Catalytically active recombinant human QSOX1; rQSOX1(AA): Catalytically inactive mutant QSOX1; shRNA: short hairpin RNA; TMA: Tumor tissue microarray

## Competing interests

The authors declare that they have no competing interests.

## Authors' contributions

BAK participated in the design, execution, analysis and interpretation of *in vitro *studies and drafting of the manuscript. ITO provided pathological interpretation of IHC results, statistical analysis and contributed to the drafting of the manuscript. HEC participated in analysis and interpretation of results and provided significant input into drafting the manuscript. YHC provided statistical evaluation of IHC results and contributed to the drafting of the manuscript. GH participated in the design and interpretation of *in vitro *results. AW and JL performed IHC on TMA samples. DL participated in the conception, design, interpretation of results and drafting of the manuscript. All authors have read and approved the manuscript for publication.

## Supplementary Material

Additional file 1 **Full Western blot and gelatin zymography images**. **a) **Western blot of MCF10A confluent, MCF10A 30% confluent, MCF7, MDA-MB-468, MDA-MB-543, BT549 and MDA-MB-231 total cell lysate probing for QSOX1 and Bactin. **b) **Western blot of MCF7 Untreated, shScramble, sh742, sh528, sh616 and sh613 total cell lysate probing for QSOX1. **c) **Western blot of BT549 untreated, shScramble, sh742, sh528 and sh616 total cell lysate probing for QSOX1. **d) **Western blot of MCF7 untreated, shScramble, sh742, sh528, sh616 and sh613 probing for alpha-tubulin. **e) **Western blot from left to right MCF7 untreated, shScramble, sh742, sh528 and sh616; BT549 untreated, shScramble, sh742, sh528 and sh616; H2O2 treated MCF7 cells, probing for alpha-tubulin. **f) **Western blot of MCF7 Untreated, shScramble, sh742, sh616 and sh528 probing for Vimentin. **g) **Western blot from left to right MCF7 untreated, shScramble, sh742, sh528 and sh616; BT549 untreated, shScramble, sh742, sh528 and sh616; H2O2 treated MCF7 cells, probing for LC3. **h) **Western blot of BT549 untreated, shScramble, sh742, sh528 and sh616 probing for Vimentin. **i) **Western blot of BT549 untreated, shScramble, sh742, sh528, sh616 and sh613 probing for alpha-tubulin. **j) **Western blot from left to right MCF7 untreated, shScramble, sh742, sh528 and sh616; BT549 untreated, shScramble, sh742, sh528 and sh616; H2O2 treated MCF7 cells, probing for caspase 3. **k) **Gelatin zymography of BT549 untreated, shScramble, sh742, sh528 and sh616. Clear bands indicated MMP-2 and -9 digestion. **l) **Gelatin zymography of MCF7 untreated, shScramble, sh742 ad sh528. Clear bands indicate MMP-2 and -9 digestion. **m) **Western blot of, from left to right, MCF7 untreated, shScramble, sh742 and sh528; BT549 untreated, shScramble, sh742 and sh528. Blot was probed for MMP-2, then stripped and reprobed for MMP-9. **n) **Western blot of ZR75, BT474 and MCF7 Untreated, shScramble, sh742 and sh528 probing for QSOX1. **o) **Western blot of BT474 untreated, shScramble, sh742 and sh528 probing for QSOX1 and alpha-tubulin. Click here for file

Additional file 2 **GOBO (Gene expression based outcome for breast cancer online) analyses of QSOX1 gene expression**. For **a-d **and **g-k**, gray line represents tumors weakly expressing QSOX1 transcript; red line represents tumors strongly expressing QSOX1 transcript. Kaplan-Meier analysis using relapse free survival (RFS) and overall survival (OS) as an endpoint for a.) All Tumors - RFS (*n *= 914); b.) All Tumors - OS (*n *= 737); c.) Untreated Tumors - RFS (*n *= 415); d.) Untreated Tumors - OS (*n *= 307); e.) Luminal B - RFS (*n *= 130); f.) Luminal B - OS (*n *= 98); stratified into five quintiles based on QSOX1 expression level. Purple line represents the highest fifth of QSOX1 expression where 50% median RFS is less than two years for RFS and less than three years for OS. g.) Luminal A - RFS (*n *= 261); h.) Luminal A - OS (*n *= 189); i.) Grade 1 - OS (*n *= 139); j.) Grade 2 - OS (*n *= 315); k.) Grade 3 - OS (*n *= 262). Click here for file

Additional file 3 **Suppression of QSOX1 in MCF7 and BT549 cells does not lead to a significant increase in apoptosis or autophagy**. **a.) **MCF7, **b.) **BT549 and **c.) **BT474 cells treated with shRNAs were analyzed for deviations in the cell cycle. Analysis was performed using propidium iodide to label DNA and analyze cells in G_1_, S and G_2_/M of the cell cycle by flow cytometry. Annexin V/Propidium Iodide analysis was performed on **d.) **MCF7 and **e.) **BT474 cells to assess apoptosis. Western blot analysis of **a) **MCF7 and BT549 untreated, shScramble, sh742 and sh528 total cellular protein probed for LC3. BT549 cells incubated exogenously with 50 uM H_2_O_2 _to induce expression of LC3 (autophagy) is used as a positive control. Full gel images can be seen in Additional file 1. Click here for file
