# Correction to: Expression of quiescin sulfhydryl oxidase 1 is associated with a highly invasive phenotype and correlates with a poor prognosis in luminal B breast cancer

**DOI:** 10.1186/s13058-018-0998-7

**Published:** 2018-08-08

**Authors:** Benjamin A. Katchman, I. Tolgay Ocal, Heather E. Cunliffe, Yu-Hui Chang, Galen Hostetter, Aprill Watanabe, Janine LoBello, Douglas F. Lake

**Affiliations:** 1 School of Life Sciences, Arizona State University, PO Box 874501, Tempe, AZ 85287-4501 USA; 2 Department of Laboratory Medicine and Pathology, Mayo Clinic Arizona, 13400 E. Shea Blvd, Scottsdale, AZ 85259 USA; 3 Department of Investigational Pathology, Translational Genomics Research Institute, 445 N Fifth St, Phoenix, AZ 85004 USA; 4 Division of Health Sciences Research, Mayo Clinic Arizona, 13208 E. Shea Blvd, Scottsdale, AZ 85259 USA

## Correction

After the publication of this work [1], an error was noticed in Fig. 4a. The micrograph image sh528 was accidentally duplicated. We apologize for this error and have replaced it with the correct figure below. This does not affect any of the interpretations or conclusions of the article.

**Fig. 4:**
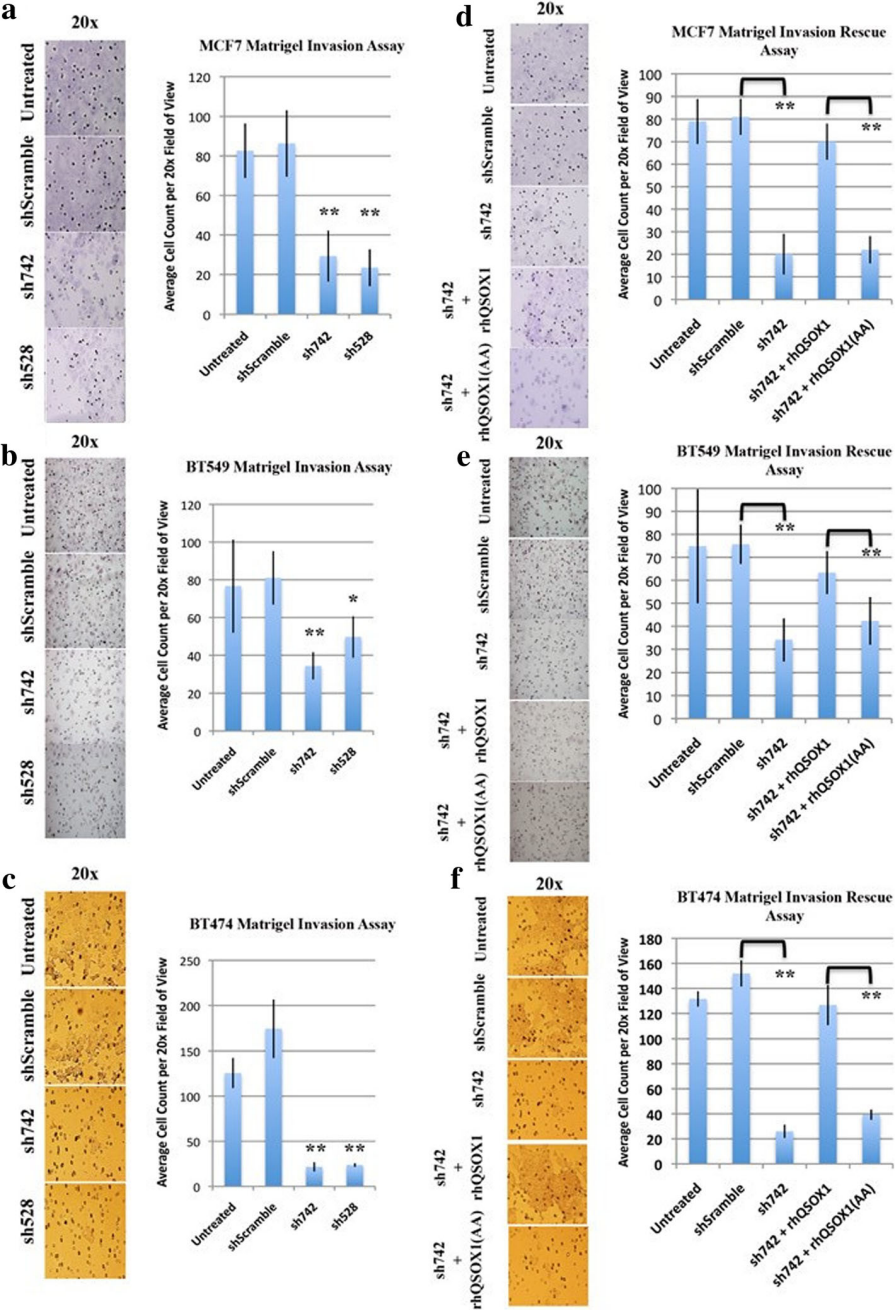
QSOX1 promotes tumor cell invasion. **a** MCF7, **b** BT549 and **c** BT474 cells transduced with shSramble, sh742 and sh528 shRNAs were seeded at equal densities in the top chamber of Matrigel™ invasion wells and allowed to incubate for 48 (BT549 and BT474) and 72 (MCF7) hours, after which cells that had digested Matrige^l^™ and migrated through the 8 um pores were counted on the underside of the insert. Representative 20× images are presented. MCF7 cells transduced with sh742 and sh528 show a 65% and 71% decrease in invasion. BT549 cells transduced with sh742 and sh528 showed a 60% and 40% decrease in invasion. BT474 cells transduced with sh742 and sh528 show an 82% decrease in invasion. Each knockdown was compared to shScramble controls. The invasive phenotype of shQSOX-transduced MCF7 (**d**), BT549 (**e**) and BT474 (**f**) cells was rescued by exogenous incubation with catalytically active rhQSOX1. rhQSOX1 (AA) mutant is a mutant without enzymatic activity, generously provided by Dr. Debbie Fass. Graphs represent average ± SD (MCF7, BT549 and BT474 *n* = 3), significance *, *P* < 0.05, ** *P* < 0.005
