# Supplementary figures and images for: Expression of quiescin sulfhydryl oxidase 1 is associated with a highly invasive phenotype and correlates with a poor prognosis in Luminal B breast cancer
Source: Breast Cancer Res. 2013 Mar 28;15(2):R28. doi: 10.1186/bcr3407 (PMC3738157; doi:10.1186/bcr3407)

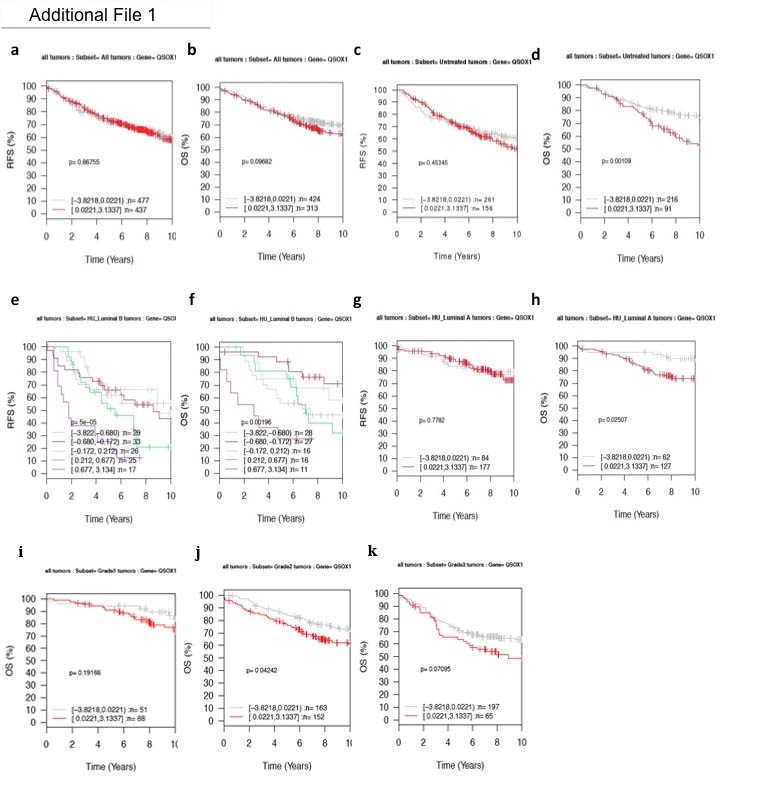

Supplement: Additional file 1 — Full Western blot and gelatin zymography images. a) Western blot of MCF10A confluent, MCF10A 30% confluent, MCF7, MDA-MB-468, MDA-MB-543, BT549 and MDA-MB-231 total cell lysate probing for QSOX1 and Bactin. b) Western blot of MCF7 Untreated, shScramble, sh742, sh528, sh616 and sh613 total cell lysate probing for QSOX1. c) Western blot of BT549 untreated, shScramble, sh742, sh528 and sh616 total cell lysate probing for QSOX1. d) Western blot of MCF7 untreated, shScramble, sh742, sh528, sh616 and sh613 probing for alpha-tubulin. e) Western blot from left to right MCF7 untreated, shScramble, sh742, sh528 and sh616; BT549 untreated, shScramble, sh742, sh528 and sh616; H2O2 treated MCF7 cells, probing for alpha-tubulin. f) Western blot of MCF7 Untreated, shScramble, sh742, sh616 and sh528 probing for Vimentin. g) Western blot from left to right MCF7 untreated, shScramble, sh742, sh528 and sh616; BT549 untreated, shScramble, sh742, sh528 and sh616; H2O2 treated MCF7 cells, probing for LC3. h) Western blot of BT549 untreated, shScramble, sh742, sh528 and sh616 probing for Vimentin. i) Western blot of BT549 untreated, shScramble, sh742, sh528, sh616 and sh613 probing for alpha-tubulin. j) Western blot from left to right MCF7 untreated, shScramble, sh742, sh528 and sh616; BT549 untreated, shScramble, sh742, sh528 and sh616; H2O2 treated MCF7 cells, probing for caspase 3. k) Gelatin zymography of BT549 untreated, shScramble, sh742, sh528 and sh616. Clear bands indicated MMP-2 and -9 digestion. l) Gelatin zymography of MCF7 untreated, shScramble, sh742 ad sh528. Clear bands indicate MMP-2 and -9 digestion. m) Western blot of, from left to right, MCF7 untreated, shScramble, sh742 and sh528; BT549 untreated, shScramble, sh742 and sh528. Blot was probed for MMP-2, then stripped and reprobed for MMP-9. n) Western blot of ZR75, BT474 and MCF7 Untreated, shScramble, sh742 and sh528 probing for QSOX1. o) Western blot of BT474 untreated, shScramble, sh742 and sh528 probing for QSOX1 a [file bcr3407-S1.JPEG]

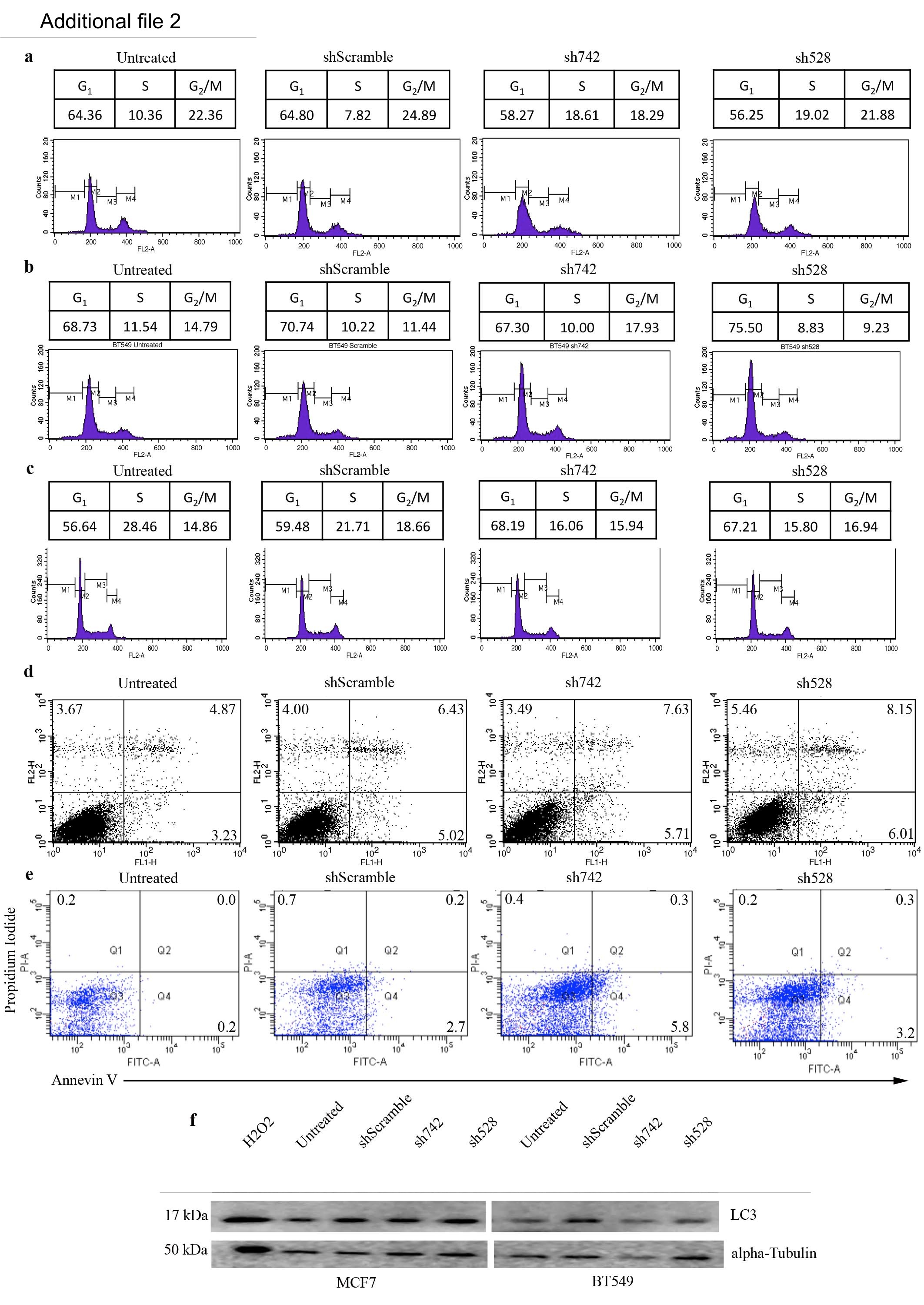

Supplement: Additional file 2 — GOBO (Gene expression based outcome for breast cancer online) analyses of QSOX1 gene expression. For a-d and g-k, gray line represents tumors weakly expressing QSOX1 transcript; red line represents tumors strongly expressing QSOX1 transcript. Kaplan-Meier analysis using relapse free survival (RFS) and overall survival (OS) as an endpoint for a.) All Tumors - RFS (n = 914); b.) All Tumors - OS (n = 737); c.) Untreated Tumors - RFS (n = 415); d.) Untreated Tumors - OS (n = 307); e.) Luminal B - RFS (n = 130); f.) Luminal B - OS (n = 98); stratified into five quintiles based on QSOX1 expression level. Purple line represents the highest fifth of QSOX1 expression where 50% median RFS is less than two years for RFS and less than three years for OS. g.) Luminal A - RFS (n = 261); h.) Luminal A - OS (n = 189); i.) Grade 1 - OS (n = 139); j.) Grade 2 - OS (n = 315); k.) Grade 3 - OS (n = 262). [file bcr3407-S2.JPEG]
